# Similarities and disparities between visual analysis and high-resolution electromyography of facial expressions

**DOI:** 10.1371/journal.pone.0262286

**Published:** 2022-02-22

**Authors:** Liraz Gat, Aaron Gerston, Liu Shikun, Lilah Inzelberg, Yael Hanein

**Affiliations:** 1 School of Electrical Engineering, Tel Aviv University, Tel Aviv, Israel; 2 Tel Aviv University Center for Nanoscience and Nanotechnology, Tel Aviv University, Tel Aviv, Israel; 3 Sagol School of Neuroscience, Tel Aviv University, Tel Aviv, Israel; 4 X-trodes, Herzelia, Israel; Tokai University School of Medicine, JAPAN

## Abstract

Computer vision (CV) is widely used in the investigation of facial expressions. Applications range from psychological evaluation to neurology, to name just two examples. CV for identifying facial expressions may suffer from several shortcomings: CV provides indirect information about muscle activation, it is insensitive to activations that do not involve visible deformations, such as jaw clenching. Moreover, it relies on high-resolution and unobstructed visuals. High density surface electromyography (sEMG) recordings with soft electrode array is an alternative approach which provides direct information about muscle activation, even from freely behaving humans. In this investigation, we compare CV and sEMG analysis of facial muscle activation. We used independent component analysis (ICA) and multiple linear regression (MLR) to quantify the similarity and disparity between the two approaches for posed muscle activations. The comparison reveals similarity in event detection, but discrepancies and inconsistencies in source identification. Specifically, the correspondence between sEMG and action unit (AU)-based analyses, the most widely used basis of CV muscle activation prediction, appears to vary between participants and sessions. We also show a comparison between AU and sEMG data of spontaneous smiles, highlighting the differences between the two approaches. The data presented in this paper suggests that the use of AU-based analysis should consider its limited ability to reliably compare between different sessions and individuals and highlight the advantages of high-resolution sEMG for facial expression analysis.

## Introduction

Facial muscles have relatively well-defined geometry and specific actions, such as pulling the lips upward, pressing the lips together, twitching the nose or furrowing the eyebrows [1]. Many actions rely on co-activation of two or more muscles, and some distant muscles are innervated by the same branches. Facial movements can be voluntary (coordinated by cortical pathways), reflexive, or automated (driven by central pattern generators located mainly in the brain stem), and as such they play an important role in medical diagnosis [2]. Facial muscles take part in a multitude of critically important tasks, such as chewing, swallowing, breathing, eye hydration and speech. Indeed, a multitude of medical conditions involve abnormal facial expressions. In Parkinson’s disease, for example, hypomimia, the reduction or loss of spontaneous facial, or the “mask face” is one of the main symptoms of the disease [3–6]. Other important examples are the Tourette syndrome [7] which is typified by abnormal facial muscle activation patterns, hemifacial spasm (HFS), facial paresis, aberrant regeneration and synkinesis [8, 9]. Facial muscle control is particularly complex, as facial expressions also have an important communicative and social role [10].

Mathematical representation of facial muscle activation is a key component in their study, so they can be quantified and systematically compared, both between individuals and between facial actions. To this end, several approaches were developed [11] including the FACEM [10] and the FACS [12] approaches. Once a quantitative framework has been developed, a transition to CV-based analysis is a natural step. Indeed, owing to their ease of use, CV-based tools have gained massive attention in mapping facial muscle activation [2, 13–15]. CV for facial expression analysis is rapid and objective and can be implemented with minimal training.

Despite the many benefits, several pitfalls are expected in using visual data to analyze facial expressions: First, it is expected that visual data and CV will be more reliable in identifying activation associated with visible body or skin movement. In scenarios involving isometric muscle activation, (muscular activation without apparent bodily movement) CV validity may be dubious [16]. Another major challenge in analyzing muscle activation with CV is in the identification of the movement source. CV analysis may be affected by crosstalk between different movements and may not be sensitive enough to distinguish, for example, between eye movements associated with the activation of the muscle segments around the eye, versus movements associated with pulling the cheeks upward [17]. When the lips are pulled up, they may lift part of the upper facial region, making the false impression that the eye muscles are active, when in fact they are not.

The difficulty in identifying facial movement source is best manifested in the study of smiles. Smiles are widely studied in behavioral psychology research as a non-verbal social communicative mechanism [12, 18, 19], important for social interaction and emotional expression. Of the many distinct smile types studied, a Duchenne smile (DS) and a non-Duchenne smile (NDS) are two of the most studied [18–21]. DSs, as recognized from the muscle stimulation experiments performed by Duchenne in the 19th century, involve the activation of both the *Zygomaticus* Major and the *Orbicularis Oculi* muscles. DSs are widely considered to reflect enjoyment and happiness, and they are commonly categorized as spontaneous, genuine smiles. A NDS, on the other hand, is expressed by the activation of the *Zygomaticus* Major alone, and is regarded as an artificial smile [20]. Several studies, however, question this paradigm, suggesting that a DS and a NDS have no particular consistent meaning [17, 22]. To distinguish between DS and NDS smiles using CV data, previous studies compared the activation intensity around the lips and eyes during a smile. When both of them are active simultaneously, the smile is classified as a DS. The exact cutoff appears to be subjective and varies from study to study, possibly contributing to some of the inconsistencies in published literature [21].

Despite its apparent advantages and widespread use, the validity of CV analysis remains unclear and only a few recent studies examined its soundness [23]. In one recent example, low-resolution sEMG was used to conclude that the Affectiva iMotions software facial expression detection performances are comparable to that of low-resolution sEMG [23]. High density sEMG offers an alternative approach to the study of facial expressions [24, 25]. Using blind source separation techniques, sEMG can be used to derive facial muscle activation maps with muscle-segment resolution [25]. Isometric activation can be readily recorded when visual-based analyses may reveal negligible movement or none at all.

The aim of the work presented in this paper is to find whether CV-based analysis of facial expressions and high-resolution sEMG provide similar mapping or are there any conspicuous differences. In particular, we want to examine whether the FACS approach, as a mathematical representation of facial muscles, is intra and inter subject stale. To this aim, we used our unique and newly developed high-resolution facial sEMG mapping approach, which for the first time allows such systematic comparison [25]. We compared the OpenFace software [26], a widely-used CV tool for Facial Action Coding System (FACS), to an ICA-based sEMG analysis of 16-channel facial electrode data. sEMG and AU sources were identified and compared to identify similarities and conspicuous gaps.

## Materials and methods

### Data collection

sEMG and video data used in this study were collected as detailed in [25]. In summary, 13 healthy volunteers (age: 31.77 ± 7.11 years; 9 females) participated in the experiment in accordance with relevant guidelines and regulations under approval from the Institutional Ethics Committee Review Board at Tel Aviv University. Written informed consent was obtained from all participants. Participants declared that they do not take antidepressants or stimulants (such as Ritalin). Otherwise, they were not physically or psychologically examined. As the aim of the study was to compare the mapping of facial expressions, the medical condition of the participants is not likely to affect the analysis but may alter responsiveness and expressivity. All participants exhibited significant response to the video presented to them. sEMG was recorded using specially designed screen-printed adhesive electrode arrays comprised of 16 carbon electrodes (4 mm in diameter). The electrode array was connected to an amplifier unit (Intan Technologies amplifier evaluation board, RHD2000). A commercial ground plate electrode (Natus Medical Incorporated; 019–409100) was used. sEMG was recorded at a rate of 3000 samples/s. The electrode array was located on the right side of the the participant’s face. Electrodes 0–2 were located near the upper part of the jaw, 3–8 covered the cheek region, 9–11 surrounded the eye and 12–15 were located above the eyebrow. Participants were laterally photographed during neutral facial expression for later analysis.

The experimental procedure consisted of four steps: a calibration step of voluntary expressions, a session of spontaneous smiles, an imitation step of different smile types and an additional repetition of the calibration session. Before initiating the preliminary calibration step, the experimenter showed the participants a sample of photographs and text of six expressions: big smile, wrinkle the nose, close the eyes, contract the eyebrows, press the lips and small smile, to assure that the participants understood the upcoming task. During the calibration sessions, each of the expressions above was presented on the screen for 3 s followed by a 3 s gap of neutral expression. Each facial expression was presented 3 times consecutively (total of 6 repetitions, 3 in each calibration session). These specific expressions were chosen as they are known to activate a large number of muscles. The spontaneous step was comprised of thirty-three short (5–39 s) videos that were presented to the participant, separated by 7 s of a blank slide. The participants were instructed to watch the videos and react spontaneously, and to perform specific facial movements when written descriptive commands were shown on the screen. This step involved three video types: 16 funny episodes, 12 individuals smiling to the camera and 5 written instructions to smile. The imitation step included 12 videos of individuals smiling spontaneously or on command. The participants were instructed to imitate the facial expressions in the videos.

### Data analysis

**Fig 1:**
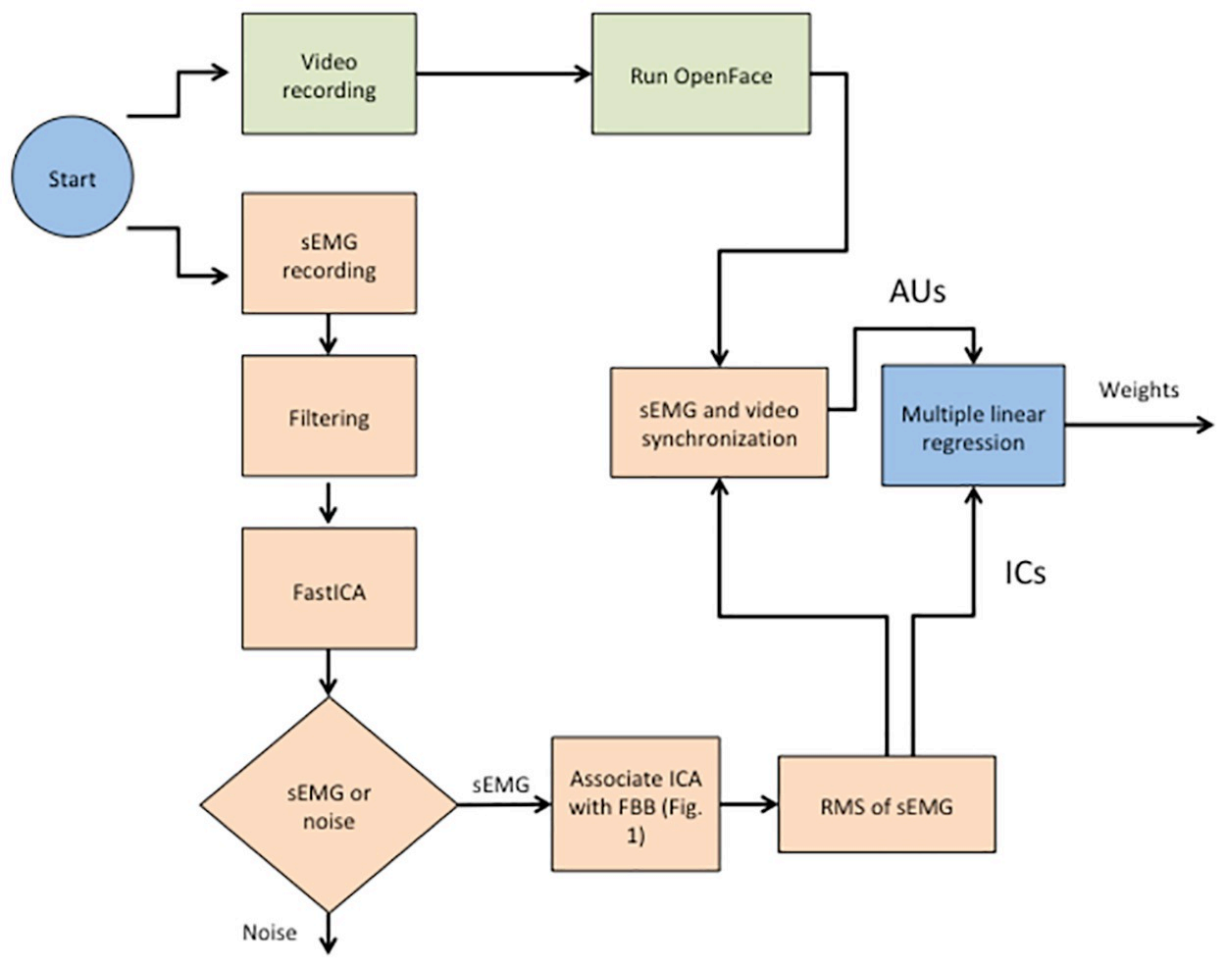
Data analysis flow chart. sEMG and video data were collected simultaneously. Video data was analysed using the OpenFace software to yield AUs. sEMG data were first filtered, separated to independent components using the fastICA algorithm, manually classified as sEMG or noise, manually associated to a specific FBB, converted to RMS and finally aligned to the AU data.

Data analysis flow is depicted in Fig 1.

#### sEMG data analysis

*Pre-processing*. Raw sEMG data was filtered using a 50 Hz comb filter to reduce the power line interference. A 20—400Hz 4th-order Butterworth bandpass filter (BPF) was applied to attenuate non-sEMG signal components.

*Independent components analysis*. Independent Component Analysis (ICA), a widely used blind source separation technique, is a computational method for separating a multivariate signal into additive independent non-Gaussian sources or components. ICA transforms the observed data *x* = [*x*_1_, ⋯, *x*_*n*_]^*T*^ into a vector of maximally independent components *s* = [*s*_1_, ⋯, *s*_*n*_]^*T*^ using a linear transformation *W*, such that *s* = *Wx*. In the case of sEMG, recorded signals from each electrode are composed of a superposition of electrical activity picked up by the electrodes from different facial muscles, external noise and mechanical and physiological artifacts. When applied to multi-channel EMG recordings, ICA has been shown to vastly improve differentiation between muscles while minimizing or entirely removing crosstalk [27]. In this analysis, the 16 recorded channels of sEMG are the observed vector *x*, while the output vector *s* is a 16-column data set, where each column represents a unique source. The sources can be either sEMG signals, noise or possibly other bio-potential signals, such as those produced by eye movements. In this investigation, we applied the fast ICA (fastICA, using the fastica function from MATLAB FastICA library) algorithm [27] to the full-length 16-channel sEMG recordings separately for each session to yield its originating signal sources or independent components (ICs). Power spectral density (PSD) was then calculated for each IC. Sources resulting from fastICA were considered to represent sEMG if they met all of the following criteria: (1) PSD was within the typical sEMG frequency range of 25–300 Hz; (2) No harmonies (periodically repeating peaks) were present; (3) PSD did not contain a strong 1/f component.

**Fig 2:**
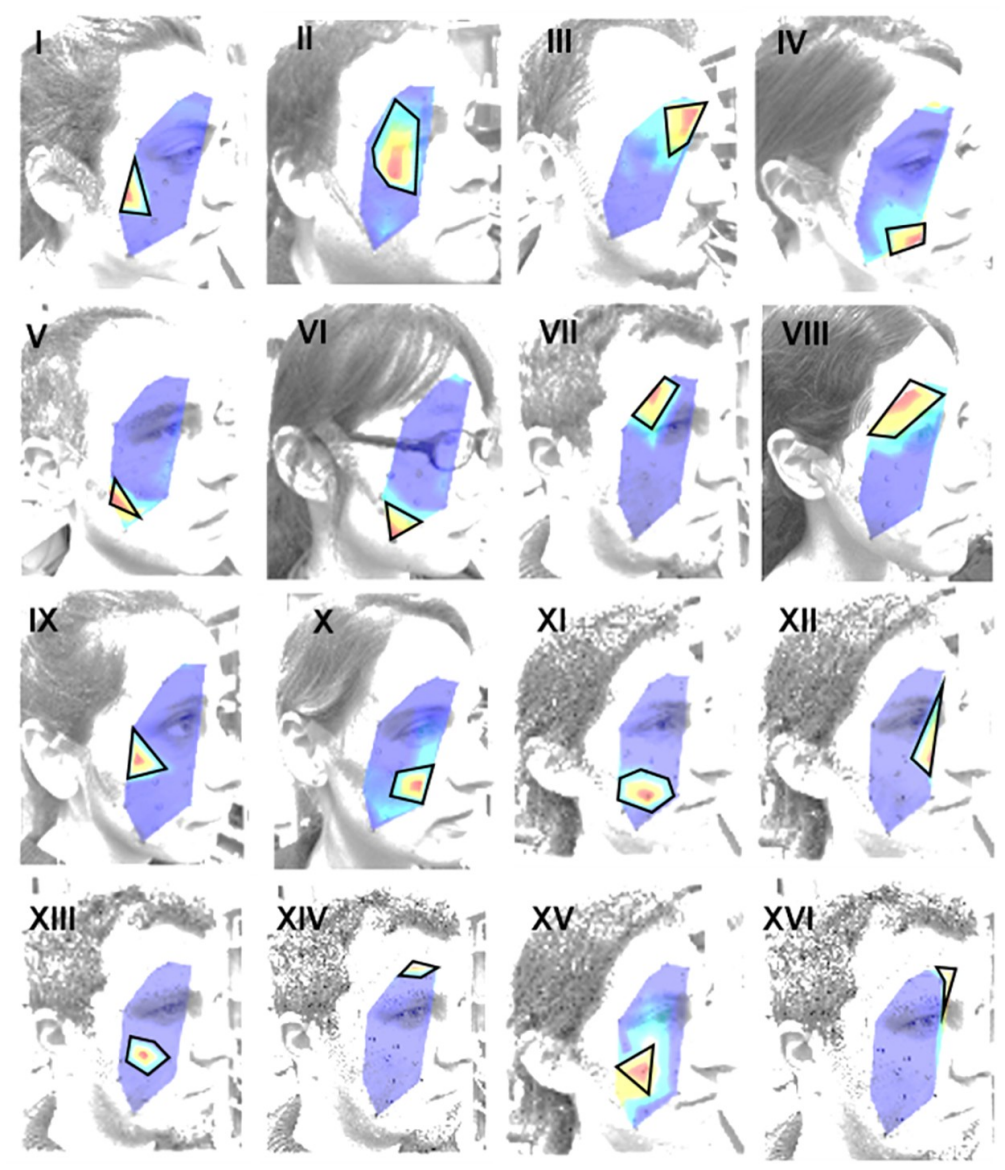
Facial building blocks. Most prominent FBBs (black contours) sketched manually on typical heat-maps, projected on lateral photographs of the participants. Red indicates highest muscle activation and blue denotes the lowest.

*ICA mapping and association with Facial Building Blocks (FBBs)*. ICA mapping builds on our previously published approach in which we used data from 13 participants to identify 16 consistent muscle and muscle-segment sources [25]. Briefly: ICs were first derived from calibration data consisting of six repetitions of instructed facial expressions (big smile, wrinkling the nose, closing the eyes, contracting the eyebrows, pressing the lips and a small smile). Each calibration set of each participant was first pre-processed and then FastICA was applied to each expression. Next, the electrode locations were used to interpolate the values of the inverse weights matrix, *W*^−^1 on the image surface to construct heat-maps. These projections reveal the physical location and shape of each IC. By grouping together ICs derived from different facial activations by the same participant and by different participants, it is possible to extract consistent facial building blocks (FBBs), which we can associate to specific muscle and muscle-segment sources based on their anatomical position. 16 FBBs identified by the ICA, based on data from 13 individuals, are shown in Fig 2, and are numbered in roman numerals from I to XVI. Red and blue denotes maximal and minimal muscle activation, respectively [25].

In this investigation, each of the IC heat maps of each session of each individual was manually classified into one of the 16 FBBs, shown in Fig 2 according to similarity in shape, size and location. ICA heat maps were derived from entire recording sessions (consisting of many expressions). The ICA outputs were renumbered according to the numbering of the relevant FBB source. Better automation can be achieved by, for example, implementing automated classification approaches [28–30].

#### Video analysis

Video analysis was performed using the OpenFace software, a commonly used tool for facial analysis [26]. This toolkit provides facial landmark detection, head pose estimation, facial AUs recognition and eye gaze estimation [26]. We focused on the AU intensity detection provided by an OpenFace built-in algorithm for automatic coding of facial expressions. The algorithm is an adapted AU recognition framework based on support vector machines [26]. All 17 AUs derived from the algorithm (AU1—Inner Brow Raiser, 2—Outer Brow Raiser, 4—Brow Lowerer, 5—Upper Lid Raiser, 6—Cheek Raiser, 7—Lid Tightener, 9—Nose Wrinkler, 10—Upper Lip Raiser, 12—Lip Corner Puller, 14—Dimpler, 15—Lip Corner Depressor, 17—Chin Raiser, 20—Lip Stretched, 23—Lip Tightener, 25—Lips part, 26—Jaw Drop, 45—Blink) were used.

#### EMG and video analysis comparison

*Data synchronization for video analysis*. sEMG and video recording were synchronized using a LabVIEW-based interface. For mathematical comparison between video analysis and sEMG, the sEMG data were down-sampled from 3000 to 30 samples/s to match the video recording sampling rate. In addition, an RMS envelope was applied to the sEMG signals using a moving window of 800 samples.

*Multiple linear regression*. Multiple linear regression (MLR) was used to quantify the similarity between the AU and the ICA data. MLR is a statistical method that aims to predict a response variable using multiple explanatory variables. MLR is an extension of ordinary least-squares (OLS) regression, and its goal is to model the linear relationship between the explanatory (independent) variables and response (dependent) variable. Given a data set (*y*_*i*_, *x*_*i*_1, ⋯, *x*_*i*_ *p*](*i* = 1)^*n*^) of (n) observations, MLR assumes that the relationship between the response variable (y) and the p-vector of explanatory predictors (x) is linear with a consideration of the error variable (*ϵ*), reflecting random noise. The model takes the following form: (*y*_*i*_ = *β*_0_ + *β*_1_ *x*_*i*1_ + *β*_2_ *x*_*i*2_ + ⋯ + *β*_*p*_ *x*_*i*_ *p* + *ϵ*), such that (i = 1, ⋯, n), where (*y*_*i*_) is the dependent variable, (*x*_*i*_) is the explanatory variables, (*β*_0_) is y-intercept (constant), (*β*_*p*_) is the slope coefficients for each explanatory variable and (*ϵ*) is the error. MATLAB built-in function regress was used to apply the model separately on AU and ICA data sets and calculate the slope coefficients for each. Statistical parameters were also calculated to evaluate the model performance: (*R*^2^) (determination coefficient), F-statistic, p-value of the F-statistic and error variance.

MLR was used to reconstruct AU signals from the ICA data. For each analyzed session, the six most relevant AUs were chosen as the model dependent variables, and all ICs were used as explanatory variables. MLR was also used to reconstruct specific IC from AUs. Here, one IC of each participant was used as the dependent variable, and all AU signals were used as the explanatory variables. In both cases, coefficient values, which were derived from the MLR analysis, were used to present the signal reconstruction. Every reconstructed signal is presented as a superposition of the explanatory variables weighted using the coefficient values.

## Results

**Fig 3:**
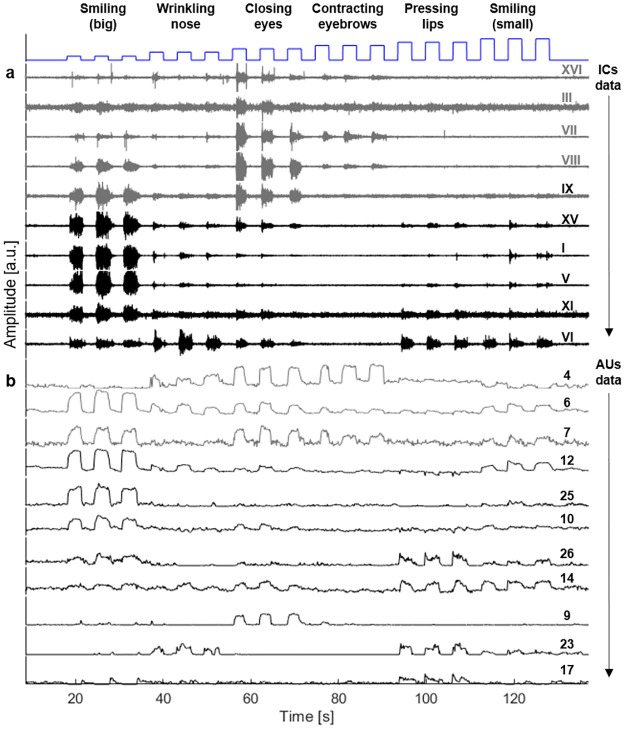
ICs and AUs data during a calibration step of participant MD8040. (a) ICs numbered according to the FBBs analysis depicted at Fig 1. (b) Typical AUs derived from video data recorded simultaneously with the sEMG recordings. Grey signals indicate muscle activation at the upper part of the face, while black, at the lower part.

We begin by comparing sEMG to CV data of one participant (participant MD8040) during six consecutive instructed expressions of the calibration step: big smile, wrinkling the nose, closing the eyes, contracting the eyebrows, pressing the lips and a small smile (Fig 3, see also S1 and S2 Figs).

Fig 3 shows IC plots numbered according to their corresponding FBBs presented in Fig 2 (a) and the AUs plots (b). AUs with an amplitude higher than 5 percent of the full scale (0–5) during at least 1 s are presented. Channels corresponding to the upper and lower parts of the face (for both panels (a) and (b)) are plotted at the top and bottom, respectively, with upper face channels plotted in grey and lower face channels in black. It is readily apparent that AUs and ICs share similar features. Foremost, they both successfully identify all six activation events (evidenced by a strong correspondence with the triggers). Second, both are generally sensitive to the origin of activation (upper versus lower parts of the face). For example, closing the eyes is captured in both IC and AU data primarily at the upper channels, while the activations corresponding to smiling (big) are contained in the lower channels.

In addition, several marked differences are also apparent and the first concerns localization. For example, during smiling (’small smile’), no activation is observed in the IC channels corresponding to activation of the upper part of the face. Besides, in the AU data, marked activity is observed in both upper and lower parts of the face. A similar effect is seen in the ‘wrinkling the nose’ activation. A second discrepancy concerns signal amplitude (muscle activation intensity) sensitivity. ICs amplitudes appear to differ from that reported by the AUs. For example, the three repetitions of ‘closing the eyes’ have different shapes (onset, offset and maximal power) in the IC data, while in the corresponding AU data, these three repetitions have almost the same amplitude. These differences between IC and AU data suggest a fundamental discrepancy between the two systems, while also raising the question whether or not these discrepancies vary between individuals or activations. To examine whether such variability exists, we used MLR to calculate the inter-relatedness between the ICA and AU systems.

### Comparison between different individuals and sessions

We first applied MLR on data from 13 different individuals that performed the calibration step, as in Fig 3, to estimate the relationship between the AUs (treated as independent variables) and a specific IC (the dependent variable). Seventeen AU signals derived from the OpenFace software were used in the model. The analysis was applied to two ICs, IC-II and IC-IX, which correspond to two well separated segments of the *Orbicularis Oculi* muscle (lower section and lower-lateral section, respectively). These segments have very robust and well identified signatures and can be readily recognized across most participants.

**Fig 4:**
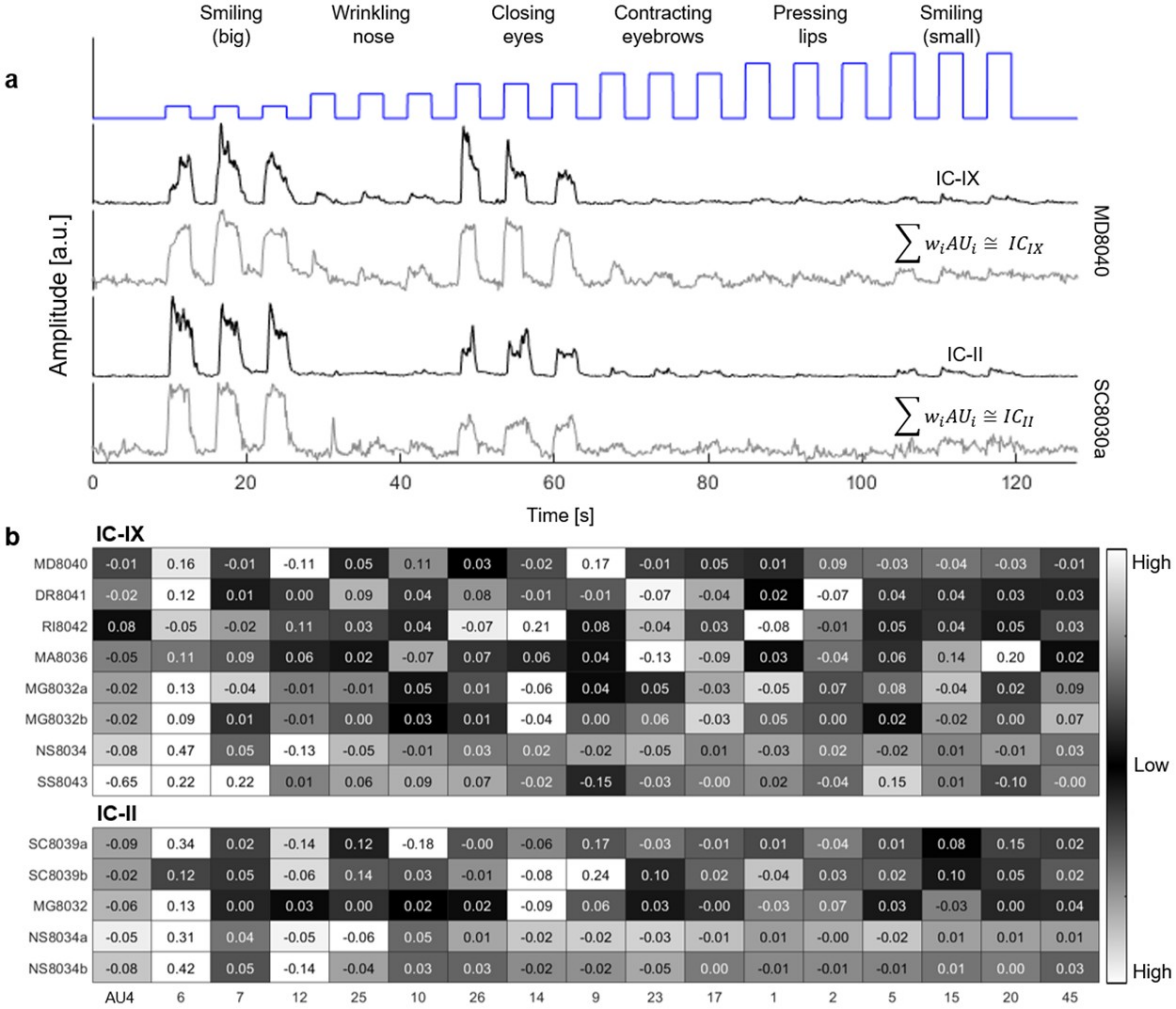
ICs data reconstruction from AUs data using multiple linear regression. (a) Top: Moving IC-IX RMS signal of participant MD8040 (black) and its reconstruction signal (grey) using MLR, where the AUs are the explanatory variables (P value (<<) 0.001, F test = 815, (*R*^2^) = 0.78, Error Variation = 0.005). Bottom: moving IC-II RMS signal of participant SC8039 and its reconstruction signal (P value (<<) 0.001, F test = 842, (*R*^2^) = 0.79, Error Variation = 0.006) (b) MLR’s weights of different participants for the reconstruction of IC-IX (top) and IC-II (bottom), color-coded in grey level: white indicates the most influential weight for a specific IC, while black indicates the lesser weight (P value (<<) 0.001, F test (>) 200, average of (*R*^2^) = 0.72, Error Variation (<) 0.016 for all MLR reconstructions).

**Fig 5:**
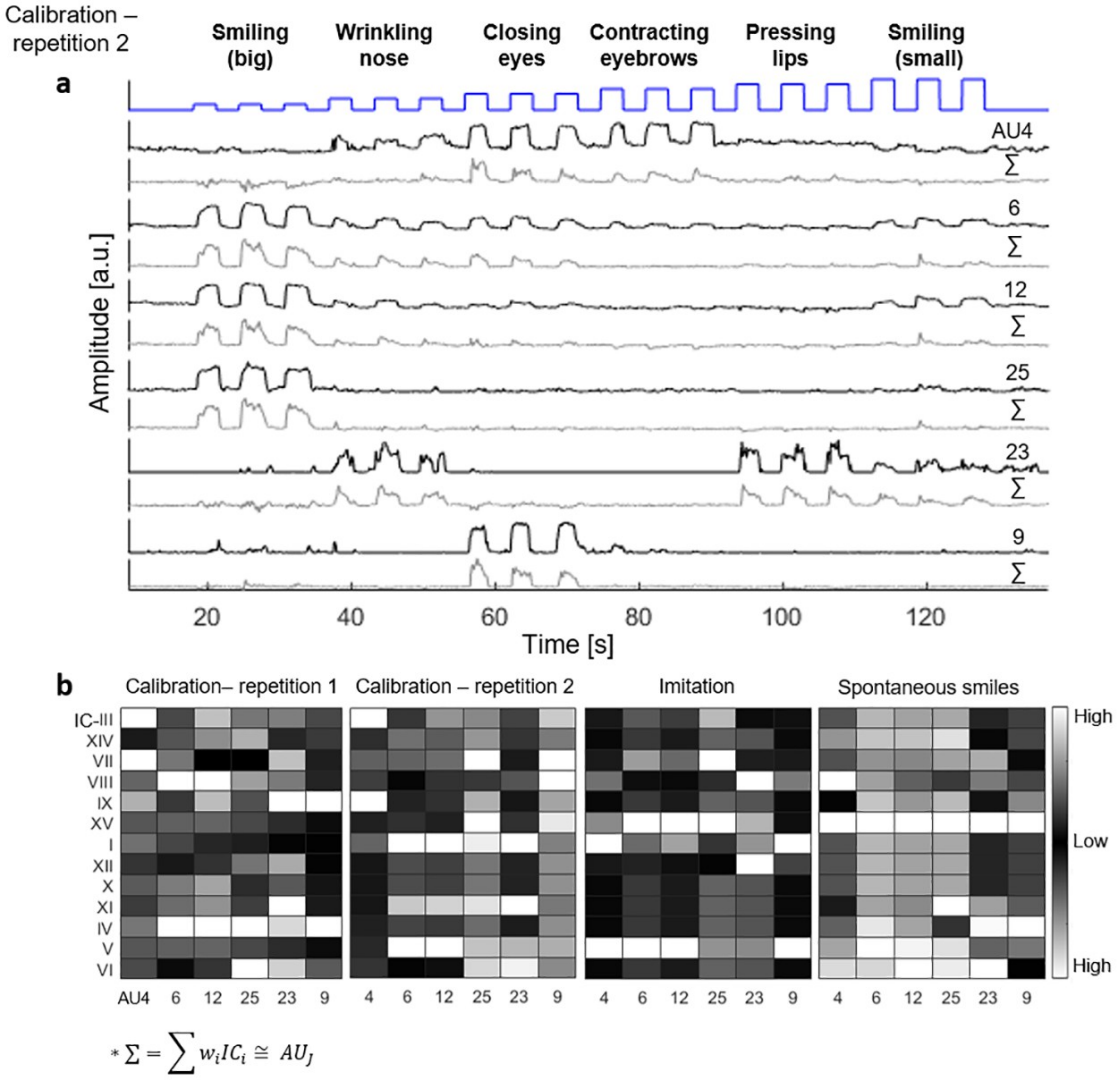
AUs data reconstruction from ICs data using multiple linear regression. (a) AUs data and the corresponding multiple linear regression reconstruction, where the ICs are the explanatory variables for a single participant during a calibration step. (b) Calculated reconstructions weights (see Methods section) for four different sessions (namely: Calibration—repetition 1 (2 min), Calibration—repetition 2 (2 min), Imitation (5 min) and Spontaneous smile (16 min) for participant MD8040), color-coded in grey level: white indicates the most influential weight for a specific AU, while black indicates the lesser weight.(P value (<<) 0.001, F test (>) 117, average of (*R*^2^) = 0.56, average of Error Variation = 0.13 for all MLR reconstructions).

Fig 4a shows two reconstructed examples calculated from data of two participants. High correlation of 0.78 and 0.79 was obtained. To quantify the quality of the reconstruction and to examine its stability across different participants, we present in Fig 4b the weights and regression metrics for IC-IX and IC-II (top and bottom, respectively). As evidenced by relatively high weights (colored white in Fig 5 tables), both IC-II and IC-IX were reconstructed primarily from AU6 (’cheek raiser’). Nonetheless, inconsistencies are common, even for two repetitions of the same session of the same participant (SC8039). For example, in three separate sessions, AU9—nose wrinkler, AU14—dimpler and AU20—lip stretcher have significant weights. These results suggest that for some participants, for some muscle activations, AU analysis results are inconsistent with ICA analysis, implying that AU analysis is participant specific.

### Intra-subject analysis

To further explore the inconsistencies described above, we reconstructed the AU data from four different sessions of the same participant (MD8040; two calibration, an imitation and a spontaneous smile sets). For each session, six of the most relevant AUs were chosen (AU4 96 brow lowerer, AU6 96 cheek raiser, AU12 96 lip corner puller, AU25 96 lips parter, AU23 96 lip tightener, AU9 96 nose wrinkler) and were reconstructed using the ICs found from the same session IC analysis.

Fig 5a presents the AU signals and their reconstruction from the ICs for one calibration session (corresponding to the session presented in Fig 2). Fig 5b shows the weight matrix for each session. Some similarities across sessions are apparent: AU23 consists mainly of IC-VI in three of the four sessions, AU4 consists mainly of IC-III in the two calibration sessions, and both smiling sessions (imitation and spontaneous) share that the reconstruction of AU6, AU12 and AU25 are derived principally from IC-XV. However, most of the weights vary, even in two similar data sets (calibration) recorded from the same participant, indicating that the superposition is not consistent across facial activations.

### Genuine versus fake smiles in video and high resolution sEMG data

The results presented above indeed suggest that AU analysis is disturbed by crosstalk. This issue may affect any facial activation analysis. We chose to illustrate this point with the analysis of smiles. One of the most debated issues regarding facial expressions concerns fake and real smiles. In particular, how and if these two smile types can be distinguished. As already demonstrated in the example shown in Fig 3, big and small smiles have a different sEMG signature. Their AU profiles, on the other hand, are similar, and can be distinguished from the lip parting component (AU25) and lip raiser (AU10). The small smile has a clear AU6 signal associated with the eyes.

**Fig 6:**
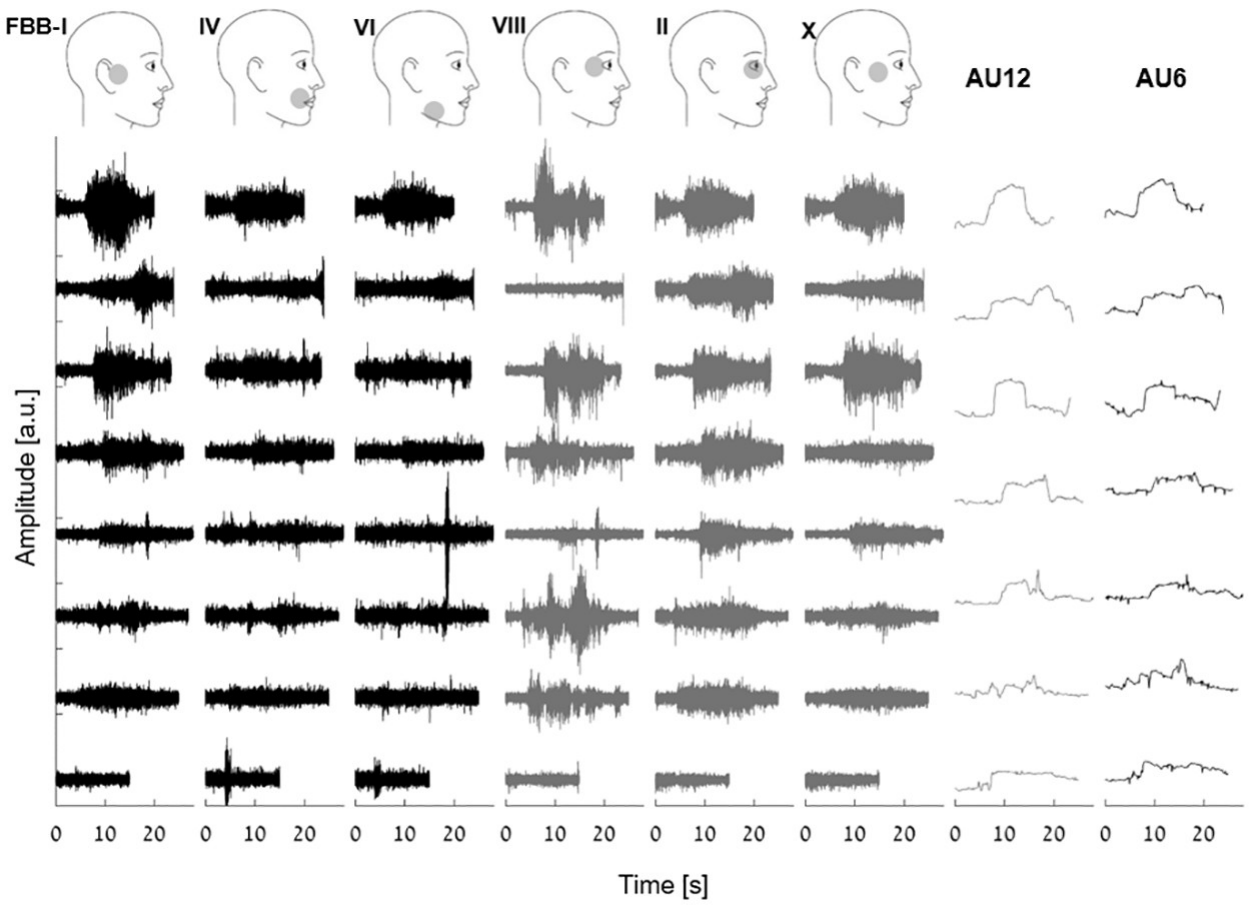
ICs and AUs data during spontaneous smiles. ICs corresponding to six specific FBBs (FBB-I, IV, VI, VIII, II and X; left) and two AUs (AU12 and AU6; right) data of eight spontaneous smiles ordered from highest (top) to lowest (bottom) amplitude (sorted according to IC-I) for participant NS8034. ICs corresponding to the upper and lower parts of the face are plotted in grey and black, respectively.

Fig 6 (see also S3 Fig) shows segmented spontaneous smiles during the spontaneous smiles session of participant NS8034. Smiles were segmented manually, according to instruction timing and SNR higher than 1.25. Each smile was confirmed with the video data. Smiles are ordered from biggest (top) to smallest (bottom) activation of IC-XIII (*Zygomaticus* muscle), according to the RMS of the signal segment. Also shown on the right are the AU6 and AU12 data of the same smiles. AU6 and AU12 appear the same with a slightly higher amplitude of AU12. In contrast, ICA data indicates variability: Some smiles are with stronger activation of the *Zygomaticus* muscle (FBBs I), and others with higher amplitude of the *Orbicularis Oculi* muscle (FBBs VIII and II).

## Discussion

Several approaches were developed to give facial expressions mathematical representation. The facial action coding system (FACS) is by far the most known and widely used. FACS describes observed facial movements and is widely used in psychological and neurological research [12]. Each AU represents contraction or relaxation of one muscle or a group of muscles, creating a specific facial movement. Identification of AU activation is based on visible movements of specific locations in the face and can be detected manually by human coders or by automatic CV algorithms.

Benefiting from high computing power, and more efficient algorithms, CV have improved dramatically in recent decades, overcoming many challenges such as sensitivity to movements and large data sets. Despite these developments, CV for facial muscle activation suffers from several fundamental shortcomings: Foremost, it does not provide direct information on muscle activation; rather, it reports on indirect physical deformations. As a consequence, it may be sensitive to crosstalk and insensitive to activations that do not involve visible deformations. Second, the use of CV in facial expressions analysis necessitates direct visual contact and high resolution, in particular for subtle and short expressions or when head movements occur.

Using a new analysis approach which builds on soft multi-electrode arrays and fastICA analysis, we were able to achieve objective mapping of facial expressions and explore the correspondence between sEMG and AU analysis across participants and sessions. In this investigation, we presented data analysis suggesting fundamental differences between high resolution sEMG mapping of facial muscle activation and CV-based facial expression estimation. Foremost, sEMG has muscle specificity and differentiation between different muscle segments. The data shown here suggests that sEMG mapping of facial muscle activation offers greater spatial resolution and is more sensitive to small changes in activation compared to AU-based analyses. AUs, on the other hand, measure facial movements that may comprise a superposition of multiple simultaneous muscle activations. Challengingly, these discrepancies may contribute to variability between participants and experiments, as we demonstrated above.

This point is especially important regarding DS and NDS classification. The standard procedure used to differentiate between these two smile types is to compare the activation power between the *Zygomaticus* and the *Orbicularis Oculi* muscles during smiling. According to this method, NDS is recognized when the *Zygomaticus* is active and the *Orbicularis Oculi* is not. Our results support the notion that the AU amplitude of the *Orbicularis Oculi* activation originates from the *Zygomaticus* muscle. In which case, NDS will be faithfully identified only in small smiles, where the *Zygomaticus* activation is low enough so there is little crosstalk to be seen around the *Orbicularis Oculi* muscle.

The duration of the data collected from each participant and number of participants in our study is limited. Although the comparison in and between participants reveals clear differences, owing to the limited sample size, we are unable to quantify the statistics of these differences. In fact, differences between individuals may be affected by many factors, such as gender, age and ethnicity. Concerning the huge body of knowledge related to the link between these and other factors and facial expressions, much more extensive mapping will be of great value and remains beyond the scope of the current investigation. Our goal here was to highlight methodological gaps which are both expected and clearly evident in the data. Moreover, our findings support previous studies which have raised similar concerns. Unlike previous reports, here we provide a physiological framework to better understand the concerns about FACS as a mathematical representation of facial expressions.

## Conclusion

AU analysis and high resolution sEMG are two alternative approaches to analyze facial expressions. The analysis we presented in this paper suggests that the AU-based approach has an inherently limited ability to reliably compare between different sessions and individuals. Reservations about the validity of CV in studying facial expressions are recognized both among computer vision specialists [31] and among investigators studying facial expressions from psychological and neurological perspectives [17, 32]. Accordingly, better algorithms and data sets continue to be developed [33]. Contemporary efforts in CV focus on identifying optimal feature representations and classifiers. Availability of extensive data sets [33] and machine learning algorithms contributed to improved reliability 6222016. Yet, several challenges remain, including the classification of the data sets themselves. It is also evident that generalization is a major challenge and there is a need for personalized facial action unit detection [34]. Despite the many concerns regarding the validity of the action unit approach, it is still widely used. In affect, research and better evidence is needed to greater highlight the shortcomings of visual analysis of facial expressions. Owing to these gaps and challenges, the significance of our results is in three domains. First, our study helps in unraveling the physiological origin for the discrepancy between CV and muscle activation. Second, for practitioners of facial action unit methods in facial expression research, we present an alternative approach, which can be readily applied for research purposes. Third, our results show that intra-subject, as well as inter-subject, are affecting CV analysis and should be considered. The discrepancies between computer vision and sEMG analysis we highlighted in our investigations are not surprising, as differences in facial features are known to impact video based facial activation mapping. The main strength of our approach is in its ability to establish an entirely objective classification of facial activation, which may be used to construct sEMG validated facial data sets in order to allow more reliable classifications which are needed in the training of new algorithms.

## Supporting information

S1 Fig ICA and CV data. (Top traces) ICA-derived signal sources containing sEMG. Associating each trace with a specific source (see Fig 1) was performed manually. (Bottom traces) AU data derived from video data recorded simultaneously with the sEMG recordings.
(TIF)

S2 Fig ICA and CV data. (Top traces) ICA-derived signal sources containing EMG. Associating each trace with a specific source (see Fig 1) was performed manually. (Bottom traces) AU data derived from video data recorded simultaneously with the sEMG recordings.
(TIF)

S3 Fig ICA and AU data during spontaneous smiles. ICA and AU data of eight spontaneous smiles ordered from highest (top) to lowest (bottom) amplitude (sorted according to IC-I).
(TIF)
