# Supplementary figures and images for: Similarities and disparities between visual analysis and high-resolution electromyography of facial expressions
Source: PLoS One. 2022 Feb 22;17(2):e0262286. doi: 10.1371/journal.pone.0262286 (PMC8863227; doi:10.1371/journal.pone.0262286)

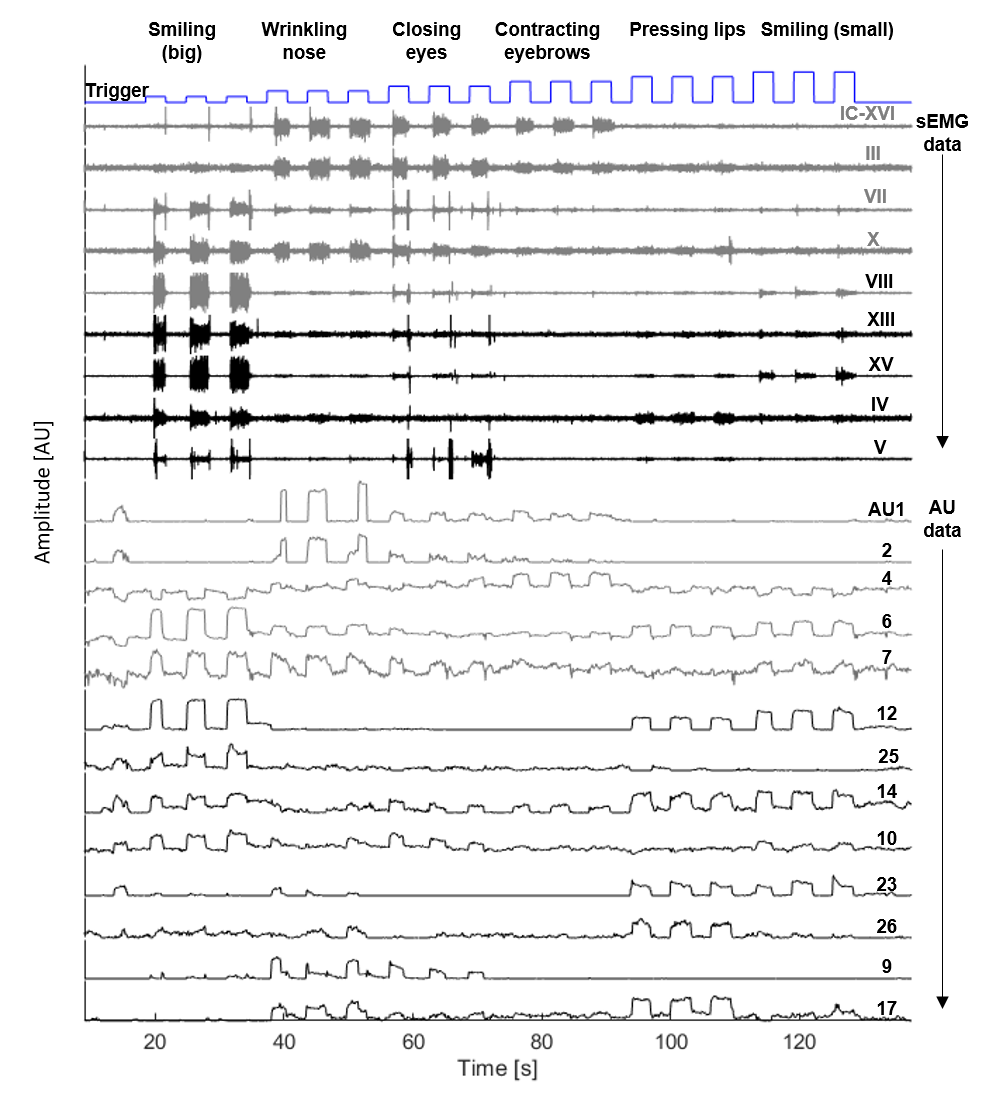

Supplement: S1 Fig — (Top traces) ICA-derived signal sources containing sEMG. Associating each trace with a specific source (see Fig 1) was performed manually. (Bottom traces) AU data derived from video data recorded simultaneously with the sEMG recordings. (TIF) [file pone.0262286.s001.tif]

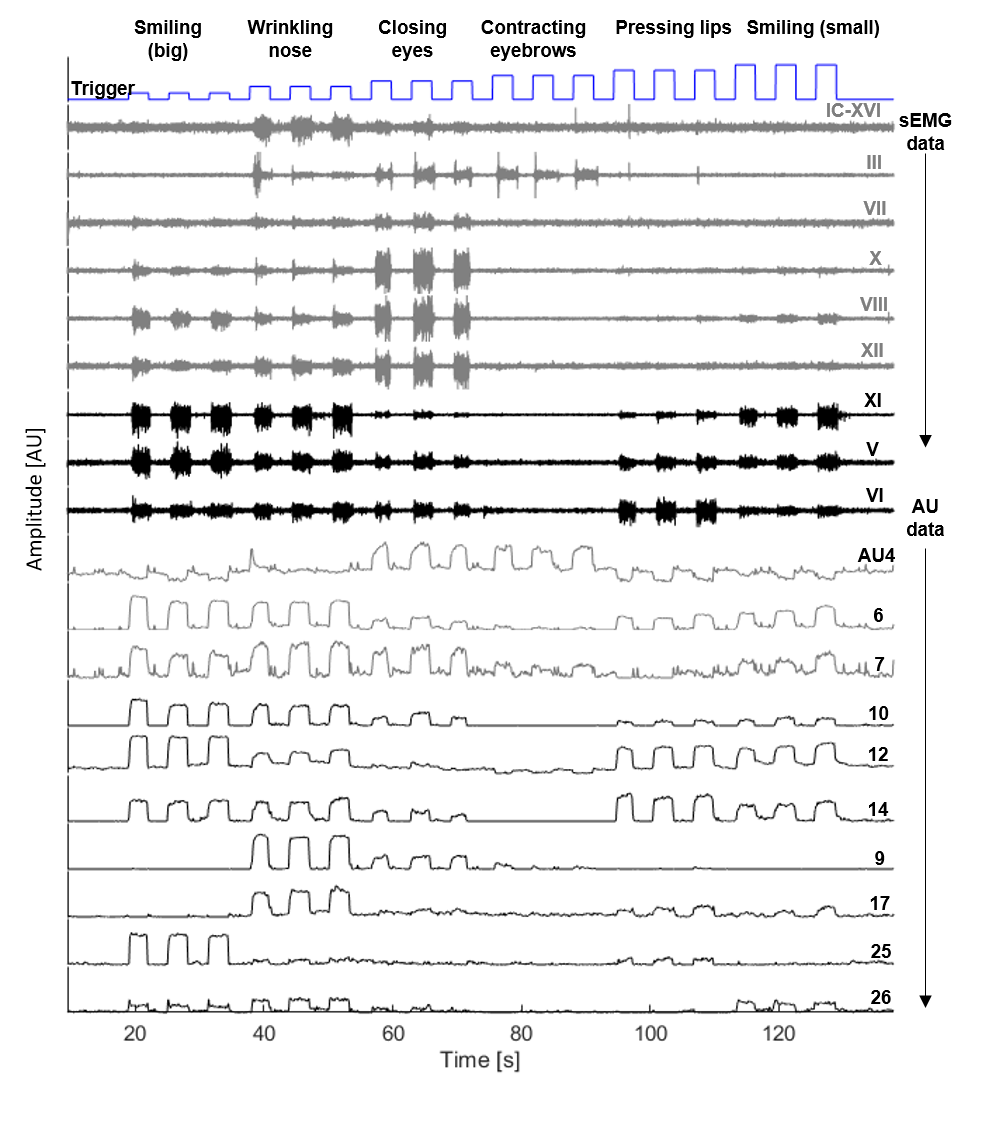

Supplement: S2 Fig — (Top traces) ICA-derived signal sources containing EMG. Associating each trace with a specific source (see Fig 1) was performed manually. (Bottom traces) AU data derived from video data recorded simultaneously with the sEMG recordings. (TIF) [file pone.0262286.s002.tif]

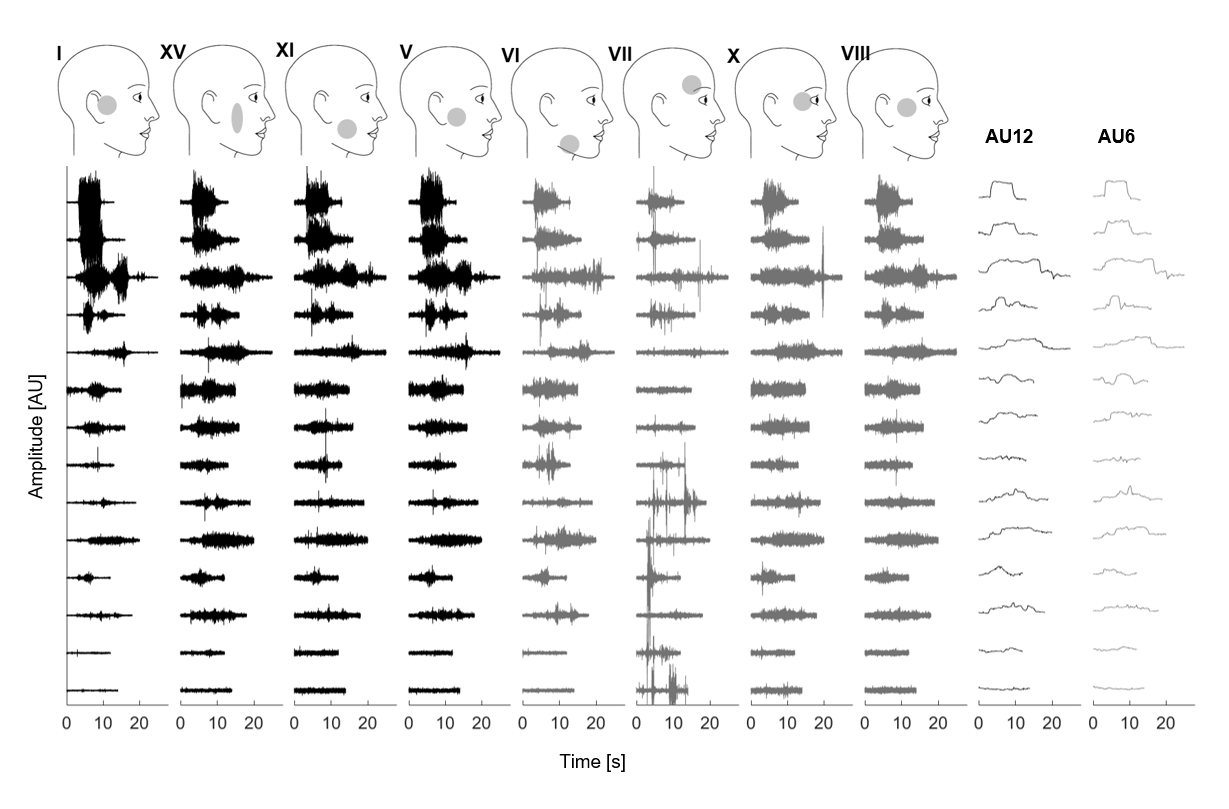

Supplement: S3 Fig — ICA and AU data of eight spontaneous smiles ordered from highest (top) to lowest (bottom) amplitude (sorted according to IC-I). (TIF) [file pone.0262286.s003.tif]
